# Undifferentiated embryonal sarcoma of the liver: a case report of a rare case in an adult patient

**DOI:** 10.1093/jscr/rjad578

**Published:** 2023-10-21

**Authors:** Ana M Marques, Guilherme Fontinha, Maria João Ferreira, Milene Sá, Júlio Constantino, Jorge Pereira

**Affiliations:** General Surgery Department, Centro Hospitalar Tondela-Viseu, Rei Dom Duarte, 3504-509 Viseu, Portugal; Pathological Anatomy Department, Centro Hospitalar e Universitário de Coimbra, Praceta Professor Mota Pinto, 3004-561 Coimbra, Portugal; Hepatobiliary and Pancreatic Department, Centro Hospitalar Tondela-Viseu, Rei Dom Duarte, 3504-509 Viseu, Portugal; Colorectal Department, Centro Hospitalar Tondela-Viseu, Rei Dom Duarte, 3504-509 Viseu, Portugal; Liver Transplant Department, Centro Hospitalar e Universitário de Coimbra, Praceta Professor Mota Pinto, 3004-561 Coimbra, Portugal; Hepatobiliary and Pancreatic Department, Centro Hospitalar Tondela-Viseu, Rei Dom Duarte, 3504-509 Viseu, Portugal

**Keywords:** undifferentiated embryonal sarcoma of the liver, hepatic sarcoma, neoplasm of the liver, surgery of de liver, case report

## Abstract

Undifferentiated embryonal sarcoma of the liver is a rare primary mesenchymal hepatic tumor that usually occurs in pediatric patients. In adulthood, this aggressive neoplasm represents only 7% of the liver sarcomas. This case reports a liver sarcoma occurring in a 49-year-old female patient. The patient was admitted in the emergency room with abdominal pain. Computerized tomography scan and magnetic resonance imaging showed a giant lobulated cystic mass in the right hepatic lobe, suggesting an atypical hemangioma. Right hepatectomy was performed. This rare case promotes a review of the differential diagnosis of liver primary neoplasms including sarcoma. The histological examination revealed an undifferentiated embryonal sarcoma. The patient underwent adjuvant chemotherapy. Currently, our patient is in complete sustained remission 4 years after chemotherapy.

## Introduction

Undifferentiated embryonal sarcoma of the liver (UESL), also called malignant mesenchymoma of the liver, is a very rare entity [1]. As its name implies, this tumor mainly occurs in pediatric patients. It is the third most common malignant tumor in older children with 75% diagnosed in aged 6–16 years [1–3]. In adulthood, it represents only 7% of the liver sarcomas and it has a higher prevalence in females between 40 and 55 years old [4].

UESL cells are poorly differentiated of undetermined cellular lineage other than mesenchymal differentiation. However, there are some sarcomas that can primarily occur in the liver and their histologic features and diagnostic criteria are almost identical to those of soft tissue tumors [1, 5, 6]. They are usually asymptomatic neoplasms; however, they can cause abdominal pain. A rare form of acute presentation is its rupture. Alpha-fetoprotein level is usually normal. UESL shows a misleading cyst-like appearance at computed tomography (CT) and magnetic resonance imaging (MRI) compared with abdominal ultrasound (US) and pathologic findings [2].

In this report we provide a literature review of an UESL in an adult patient.

## Case report

A 49-year-old female, without relevant medical history, was admitted in the emergency room with abdominal pain. The ultrasound performed revealed a nodular formation in the right hepatic lobe.

**Figure 1:**
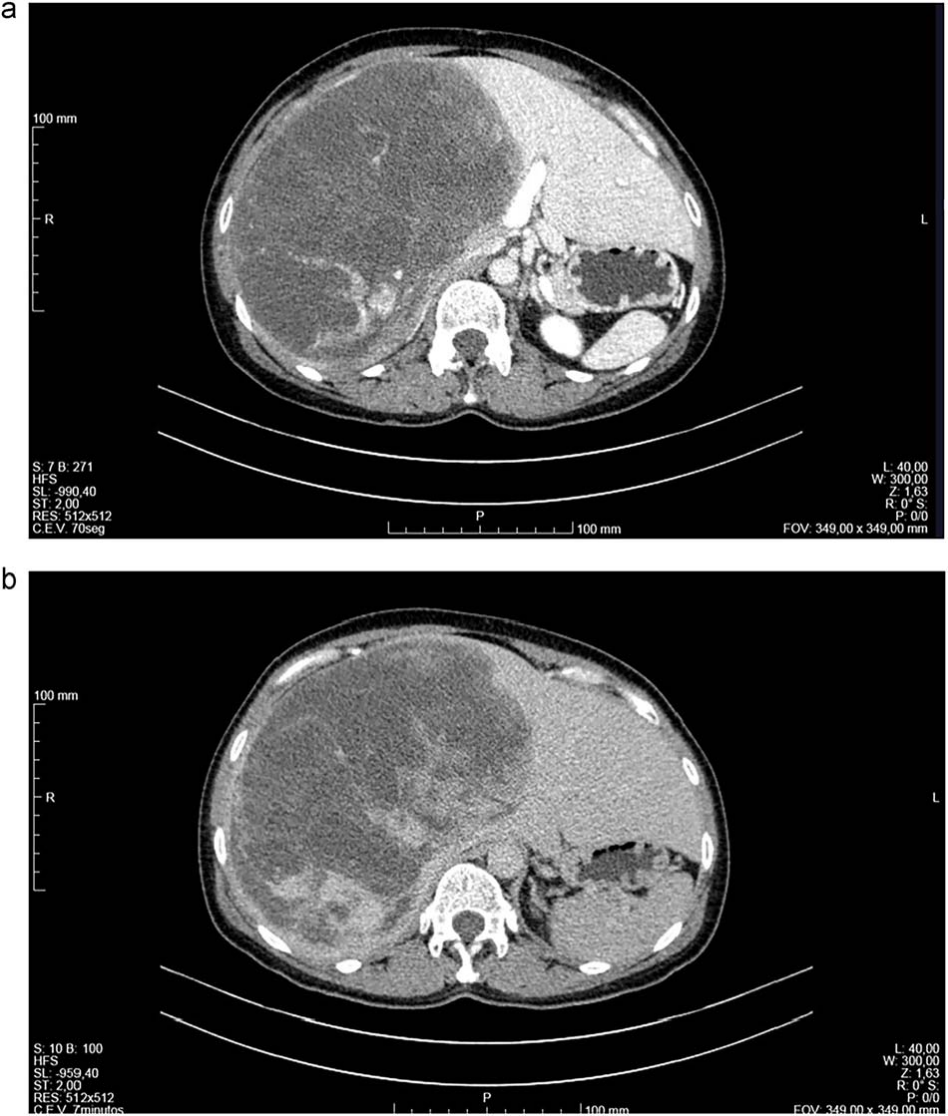
(a, b) Computed tomography (CT) scans of a woman aged 49 years with undifferentiated embryonal sarcoma of the liver. Liver of increased dimensions due to the presence of voluminous oval mass and well-defined limits occupying practically the entire right lobe (measures 16 × 16 × 20 cm^3^). It is a heterogeneous mass, with areas of apparently liquid spontaneous density. Captures contrast gradually and progressively.

**Figure 2:**
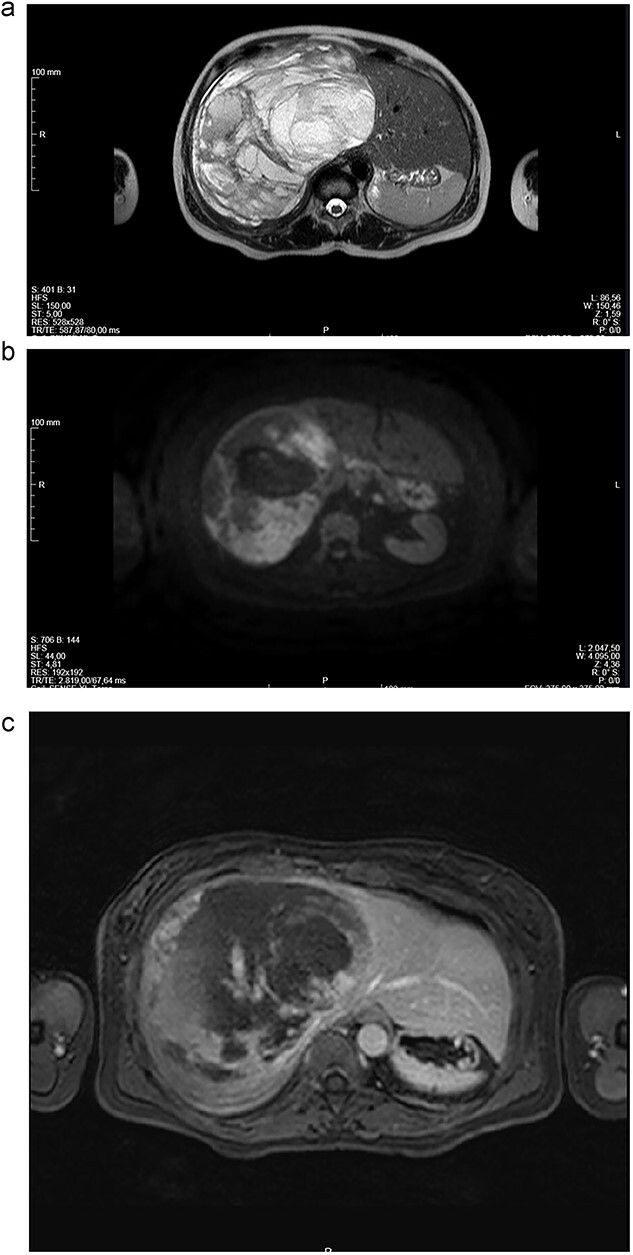
(a-c) Magnetic resonance imaging (MRI) scans of a woman aged 49 years with undifferentiated embryonal sarcoma of the liver. Liver of increased dimensions, due to the presence of a voluminous oval mass and well-defined limits occupying the entire right lobe. Heterogeneous mass with enhanced thin septa in the right lobe of the liver with fluid levels suggestive of atypical hemangioma. The right hepatic vein not dissociable to the tumor.

Abdominal CT (Fig. 1a and b) and MRI (Fig. 2a and b) showed a 18 × 12 × 20 cm^3^ lobulated cystic mass with thin enhancing septa in the right lobe of the liver with fluid levels suggesting atypical hemangioma. The right hepatic vein not dissociable to the tumor (Fig. 2c).

Liver function unchanged and serum levels of tumor markers (carcinoembryonic antigen, CA19-9, and alpha-fetoprotein) were normal. The viral markers were negative.

Given the symptomatology, dimensions and potential risk of complications (spontaneous rupture with hemoperitoneum), surgical treatment was proposed. Right hepatectomy was performed since the right hepatic vein not dissociable to the tumor (Fig. 2c).

**Figure 3:**
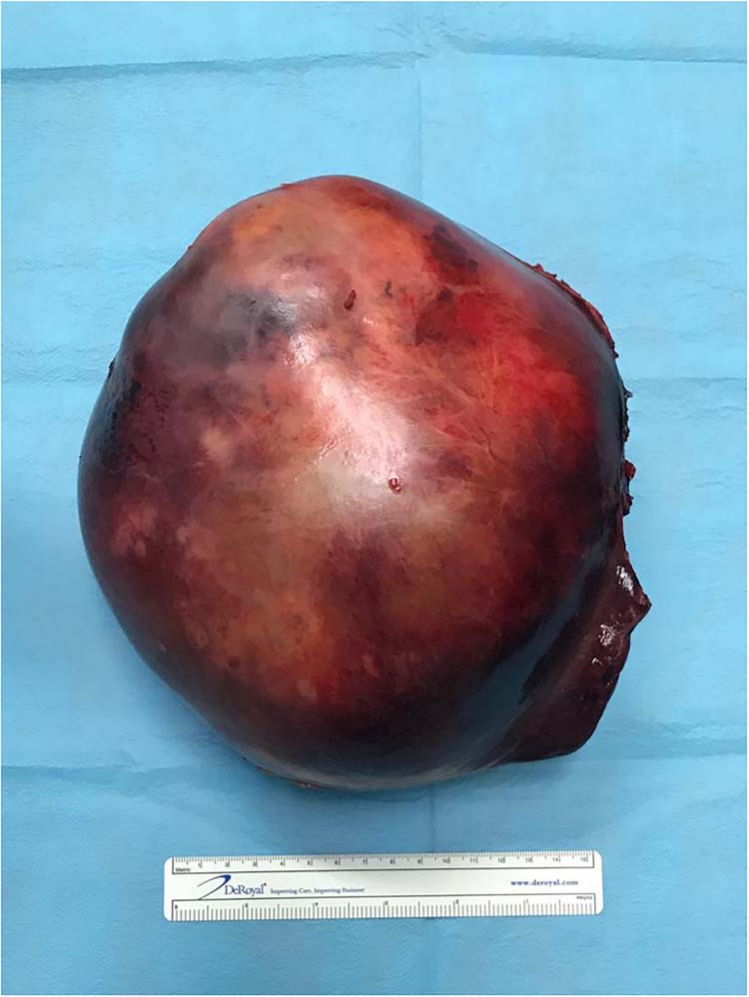
Sarcoma with a maximum diameter of a 20 cm.

The surgery elapsed without incidents (without violation of the tumor) (Fig. 3) and the patient was clinical discharged on the 7th postoperative day.

**Figure 4:**
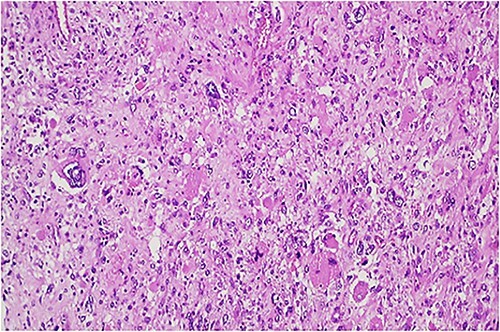
Hypercellular tumor showing severely pleomorphic neoplastic cells with occasional multinucleation.

**Figure 5:**
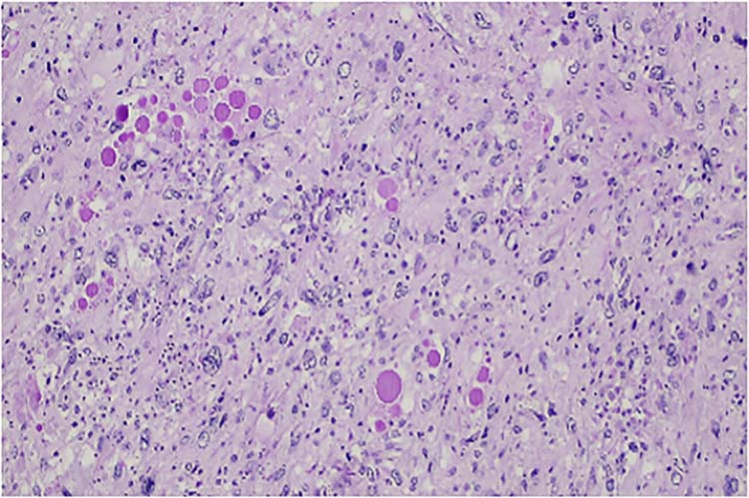
PAS-diastase positive hyaline globules are a main feature of embryonal sarcoma of the liver.

The histologic examination revealed an embryonal sarcoma (16 × 14.5 cm^2^) of the liver—a well circumscribed neoplasia with hyaline paucicellular areas and hypercellular areas (Fig. 4). These areas presented mainly spindle cells with severe pleomorphism as well as frequent mitotic figures, some of them atypical. Multinucleated giant cells with periodic acid-Schiff (PAS)-diastase positive hyaline globules were a habitual feature (Fig. 5) [7, 8]. Coagulative necrosis was present making up 20% of the tumor [2, 7, 8].

The neoplastic cells were diffusely positive for vimentin with heterogenous, focal or weak positivity for caldesmon, CD10, and smooth muscle actin. There was no staining for desmin, AE1/AE3, Cam 5.2, CD34, melan-A, S100 protein, and DOG-1 [5, 9]. In the hypercellular areas, Ki67 staining was present in more than 70% of the cells [4]. Negativity for keratins excludes hepatocellular carcinoma [2]. Other precluded entities that can be found in the liver with more or less frequency and have some concurrent histological features are angiomyolipoma (melan A, S100 protein), melanoma (melan A, S100 protein), gastrointestinal stromal tumor (DOG1), epithelioid haemangioendothelioma (CD34), and angiosarcoma (CD34) [2]. Usually in leiomyosarcoma at least two smooth muscle markers are diffusely positive. In this case there was only weak staining for smooth muscle actin and heterogenous staining for caldesmon, which are reported staining patterns for embryonal sarcoma of the liver that may prompt a wrong diagnosis [2, 10].

Colonoscopy, upper GI endoscopy and FDG PET/CT scan were performed with no alterations. Case was discussed in a multidisciplinary meeting and the patient started adjuvant chemotherapy (vincristine, actinomycin D, and cyclophosphamide) 3 months after surgery. The patient completed six cycles without complications and is alive and well, with no signs of recurrence, 4 years after the chemotherapy.

## Discussion

UESL is an aggressive and uncommon primary mesenchymal tumor that most commonly affects the pediatric patients, with a peak incidence between ages 5 and 10 years. Very few cases are reported in adult patients and can pose a considerable diagnostic challenge. Most of the time, clinical presentation is nonspecific and the radiologic exams frequently show a solid and cystic liver mass.

No specific serum markers identify UESLs, which may not have the same appearance in all radiologic exams. CT demonstrates a well-defined hypodense mass with internal septations, almost cystic with progressively enhance after contrast administration. MRI demonstrates low intensity signal in T1 and high in T2, again with enhancement on contrast administration**.**

Nowadays, the etiology of undifferentiated embryonal sarcoma of the liver is still unknown. Molecular and cytogenetic aberrations found in some UESLs include alterations in p53 tumor suppressor gene leading to inactivation, and translocation t(11;19)(q11;q13.3/13.4) and add(19)(q13.4).

The definitive diagnosis require a thorough anatomopathological analysis. UESL is characterized by high-grade undifferentiated cells with varying degrees of spin-dling and myxoid change. Like its macroscopic gross appearance, this tumor is commonly heterogeneous, and the morphology will vary considerably. Pathologic findings show that this tumor cells have a highly proliferative phenotype with equally high pleomorphism, hyperchromasia, and high rate of mitosis. Usually, this clinical entity has multiple eosinophilic blood cells that are positive for PAS and diastasis-resistant PAS [5, 7, 8]. Immunohistochemically, there is a lot of variability.

UESL is a diagnosis of exclusion. The most reported differential diagnoses are hydatid cyst, cystic tumor hemorrhage, hepatic abscess, peri-hilar cholangiocarcinoma, sarcomatoid hepatocellular carcinoma, and other liver sarcomas and mesenchymal tumors [5, 11]. This way, all other diagnostic possibilities must be excluded before this diagnosis can be made with certainty. Here comes the importance of a careful histological examination and a comprehensive immunohistochemical panel.

Nowadays, treatment includes chemotherapy in combination with surgery. This approach has been recognized as being a major factor in improving patient outcomes [2, 3]. Because a standardized approach has yet to be agreed upon, chemotherapy regimen is not consensual. About the role of radiotherapy, the effectiveness of is not clear [2, 12]. In unresectable tumors, orthotopic liver transplant can be considered [2, 12].

Due to the low incidence of UESL, especially among adults, limited data are available regarding the prognosis in these patients. However, in case reports described in the literature prognosis for UESL has been considered dismal with worse outcomes reported in adults. Factors that have been associated with improved survival are margin negative resection, receipt of chemotherapy and childhood [13]. In this case the patient received adjuvant chemotherapy after complete resection and has no evidence of disease recurrence until this date. This case presented as an atypical hemangioma in the liver and had typical histological features of UESL. It is a rare case of a common malignant pediatric tumor occurring in an adult patient.
